# Growth Modulation for Knee Coronal Plane Deformities in Children With Nutritional Rickets: A Prospective Series With Treatment Algorithm

**DOI:** 10.5435/JAAOSGlobal-D-19-00009

**Published:** 2020-01-06

**Authors:** Tamer A. EL-Sobky, Shady Samir, Mostafa M. Baraka, Tamer A. Fayyad, Mahmoud A. Mahran, Ahmad S. Aly, John Amen, Shady Mahmoud

**Affiliations:** From the Division of Paediatric Orthopaedics, Department of Orthopaedic Surgery, Faculty of Medicine, Ain-Shams University, Cairo, Egypt.

## Abstract

To report prospectively the radioclinical outcome of guided growth surgery for coronal plane deformities around the knee in young children with nutritional rickets on the intermediate term, to assess the responsiveness of torsional deformities of the tibias to guided growth regarding function and objective clinical parameters, and to propose a treatment algorithm.

**Methods:**

Fifty children (male:female, 27:23) with knee coronal plane deformities (knees:physes, 86:99), (varum:valgum, 51:35) secondary to nutritional rickets were subjected to femoral and/or tibial temporary hemiepiphysiodesis using a two-hole 8-plate. The mean age at implantation was 3.8 ± 1.5 years (range 2.5 to 5). The mean follow-up was 2.8 years (range 2 to 4). All children received a standing full-length AP radiographs of both lower limbs in neutral rotation to measure the mechanical axis deviation, tibiofemoral angle, and joint orientation angles. Tibial torsion was objectively assessed by measuring the bimalleolar axis.

**Results:**

The radiologic measurements, tibiofemoral angle, mechanical axis deviation, mechanical lateral distal femoral angle, medial proximal tibial angle, and Hilgenreiner-epiphyseal angle, showed a highly statistically significant improvement (*P* ≤ 0.001). Radiographic outcomes correlated with their clinical counterparts. The mean duration of correction of the mechanical axis was 10.8 ± 2.4 months (7 to 21). The mean follow-up for rebound of the deformity was 1.5 years (range 1 to 3).

**Conclusion:**

The radioclinical outcome is rewarding with a tolerable complication profile. The mechanical complications were mostly related to lengthy implant retainment encountered in severe deformities. Internal tibial torsion seems profoundly responsive to correction of coronal plane deformity. And, derotation osteotomies are rarely justified. Our proposed algorithm may be used as a decision-taking guide for achieving the desired growth modulation in a more efficient manner.

Guided growth surgery for lower-limb deformities took the place of the time-honored corrective osteotomies. Its effectivity and safety have been generally acknowledged.^1^ It is widely accepted that pediatric orthopaedic surgeons are supposed to use corrective osteotomies only as a salvage of a failure of guided growth surgery.^1,2^ Temporary hemiepiphysiodesis has been practiced for correction of angular knee deformities in a wide spectrum of etiologies including idiopathic,^1,3,4^ osteochondrodysplasias,^5,6^ post-traumatic,^1^ renal osteodystrophy,^7^ hypophosphatemic rickets,^8^ and arthrogryposis,^9^ Temporary hemiepiphysiodesis is done using staples,^10,11^ a regular^1,10-12^ or hinged^13^ tension band plate, percutaneous transphyseal screw,^4,14^ and a one-third tubular plate.^15^ Several clinical and animal studies have shown that the above implants are equally effective and safe in achieving guided growth with marginal differences related to complication rates, surgical times, cost, and quality of life.^1,4,10,14,15,16,17,18,19,20^ Contrastingly, the response of the physis varies with age, the nature of the disease, and nutrition and can be unpredictable.^1,12,18,21,22^ Nutritional rickets in young children remains a considerable and prevalent public health problem worldwide including the developed countries.^23-25^ The orthopaedic manifestations of rickets can result in grave deformities of all extremities, gait abnormalities, and entail an increased risk of sustaining fractures.^23-25^ Nonetheless, no exclusive or prospective studies have been conducted to evaluate the performance of guided growth in knee deformities in nutritional rickets. We postulate that physeal growth modulation surgery would be successful in restoring coronal alignment of the knee. And, we hypothesize that tibial varus is a product of coronal and transverse plane malalignment. Hence, correction of tibial varus and internal torsion will occur simultaneously. The objective of this study is to report prospectively the clinical and radiographic outcome of guided growth surgery for coronal plane deformities around the knee in young children with nutritional rickets on the intermediate term. We present the largest and most homogenous series of such patient subset in the literature. In addition, we aim to assess the responsiveness of torsional deformities of the tibias to the guided growth surgery with respect to function and objective clinical parameters and finally to propose a treatment algorithm to guide the decision-making process.

## Methods

We conducted a prospective interventional observational case study at the authors' institution from February 2015 to March 2017. During the study period, 50 children with knee coronal plane deformities secondary to nutritional rickets fulfilled the inclusion criteria and were subjected to femoral and/or tibial temporary hemiepiphysiodesis using a two-hole eight-plate. The patient and deformity characteristics are displayed (Table 1). Rickets was diagnosed when children possessed clinical skeletal deformities in association with characteristic radiographic physeal-metaphyseal changes and abnormalities in laboratory bone mineral profile. Rickets was considered healed based on the restoration of a normal phyeal line and radiographs and normal serum levels of calcium, phosphorus, and alkaline phosphatase. Alkaline phosphatase is an important and affordable screening tool for nutritional rickets^25^ We considered values above the cutoff (450 IU/L) as abnormal. Genu valgum and genu varum deformities were included. The general inclusion criteria were children younger than 7 years and healed nutritional rickets. We considered patients indicated for surgery if they possessed one or more of the following criteria: (1) an overall coronal plane deformity “varus or valgus” ≥ 20° as per tibiofemoral angle (TFA) that is persistent or worsening over the past 6 months, (2) a mechanical axis deviation (MAD) bisecting the knee outside the central one-third of the transverse diameter of the proximal tibial epiphysis, and (3) a clinically symptomatic gait impairment in the form of troublesome circumduction gait in association with genu valgum or massive intoeing in association with genu varum or frequent falls. Exclusions were (1) previous guided growth surgery or osteotomy on the affected limb, (2) osteochondrodysplasias, endocrinopathies, Blount disease, post-traumatic/infectious deformities, and (3) all forms of resistant rickets. The study was approved by our institution's Ethical Committee of Scientific Research.

**Table 1:** Patient and Deformity Characteristics

Total participants	50 children
Age (yr)	Mean: 3.8 ± 1.5 (2.5-5)
Sex	
Males	27 (54%)
Females	23 (46%)
Side	
Left	11 (22%)
Right	3 (6%)
Bilateral	36 (72%)
Deformity (no. of limbs/knees)	
Varum	51 (59%)
Valgum	35 (41%)
Total	86 knees
No. of instrumented physes	
Femur	30
Tibia	39
Biphyseal	30 (in 15 knees)
Total	99
Mean follow-up (yr)^a^	2.8 years (range 2-4)
Mean follow-up for rebound^b^	1.5 years (range 1-3)
Mean duration of correction (mo)	10.8 ± 2.41 (7-21)

a The mean follow-up from implantation up to the last visit.

b The mean follow-up from explantation to the last visit.

### Surgical Technique

We approached the involved physis directly maintaining dissection extraperiosteal. We used fluoroscopy to avoid epiphyseal plate and joint line injury while maintaining parallelism of screws and their placement in midlateral plane as much as possible. A thin Kirschner wire was inserted in the central hole of the eight-plate to mark the physis. A 2.5-mm drill bit was used to insert a 3.5-mm solid screw. The epiphyseal screw was inserted first. The drill bit was advanced for only a few millimeters into epiphyseal bone, and then, the screw was tightened till it fitted into the corresponding hole. Screw length was selected so as to reach at least the midline of the epiphysis.

### Outcome Measures

**Figure 1:**
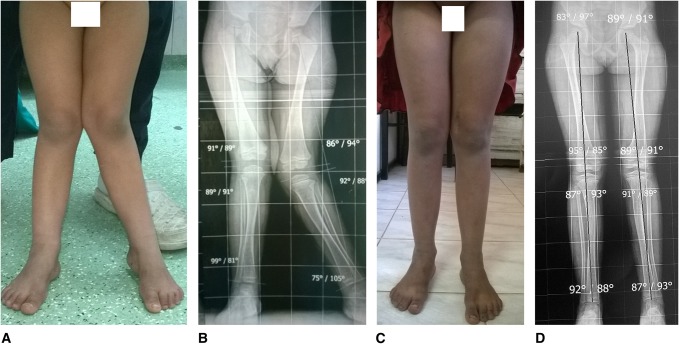
**A**–**D**, Guided growth for a 4-year-old girl with unilateral genu valgum. **A** and **B**, Preoperative clinical and radiologic findings. Note the mechanical axis is traversing zone 3. **C** and **D**, Radioclinical outcome at 3.6-year postimplant insertion. Note the mechanical axis is optimally corrected, i.e., traversing the lateral half of the central one-third of the widest diameter of the proximal tibia.

We conducted a full screening orthopaedic examination with observational gait analysis. We have done a goniometric measurement of the standing TFA and the transmalleolar axis in the prone position as an indicator of tibial torsion. We obtained standing full-length AP radiographs of both lower limbs in neutral rotation directly before the index surgery. The standing long radiograph was repeated once again once the limbs were clinically straightened before plate removal. The radiographic indices calculated were TFA, MAD, mechanical lateral distal femoral angle (mLDFA), medial proximal tibial angle (MPTA), and Hilgenreiner-epiphyseal angle (HEA).^26^ The TFA was defined as the angle between the following two lines. The first line is between the center of proximal diaphysis of the femur and the center of distal diaphysis of the femur, and the second line is between the center of proximal diaphysis of the tibia and the center of distal diaphysis of the tibia. The MAD was obtained by measuring the distance between mechanical axis and midpoint of tibial epiphysis expressed as percentage to ½ of the width of tibial epiphysis. This measurement of MAD would eliminate errors arising from magnification on radiographs and augment validity of results. Deformity severity was graded into four zones by measuring the distance between mechanical axis and midpoint of tibial epiphysis expressed as percentage to 1/2 of width of tibial epiphysis (Figure 1). We allowed immediate weight-bearing according to tolerance. Patients were observed at weekly intervals in early postoperative period until full functional recovery and full range of motion were achieved. Otherwise mothers were instructed to do range-of-motion exercises at home. Patients were assessed clinically at 3-monthly intervals thereafter. When the coronal plane alignment of the lower extremity was deemed clinically satisfactory, standing full-length AP radiographs of both lower limbs were obtained. The end point for plate removal was identified once the mechanical axis falls within the central one-third of the tibial plateau for both varus and valgus deformities. For both varus and valgus deformities, an accepted correction equated to a mechanical axis bisecting the medial half of the central one-third of tibial plateau and an optimal correction equated to a mechanical axis bisecting the lateral half of the central one-third. We assessed the amelioration of tibial torsion objectively by measuring the bimalleolar axis. In addition, we took history of frequent falls, child's ability to participate in normal daily activity, and more vigorous activities associated with young children. We assessed rebound clinically because of logistic challenges. We defined rebound as a deviation of >10° from the clinical TFA recorded right after implant removal.

### Data Management and Analysis

The collected data were coded and tabulated using Statistical package for Social Science (SPSS 20 IBM). Descriptive statistics included the mean, SD, and range for parametric numerical data, while median and interquartile range for nonparametric numerical data. Frequency and percentage of nonnumerical data were reported. Analytical statistics included the Student *t*-test was used to assess the statistical significance of the difference between two study group means. The Mann-Whitney test (*U* test) was used to assess the statistical significance of the difference of a nonparametric variable between two study groups. Paired *t*-test was used to assess the statistical significance of the difference between two means measured twice for the same study group.

## Results

**Figure 2:**
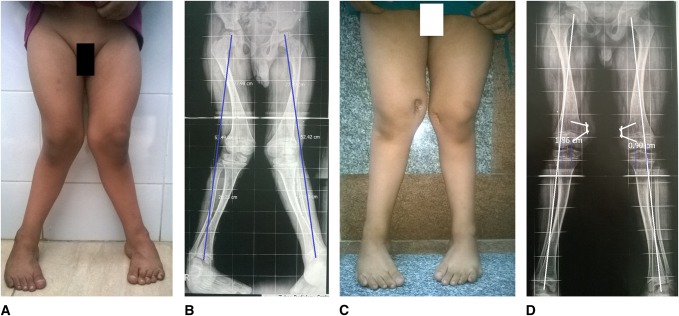
**A**–**D**, Guided growth for a 5-year-old boy with bilateral genu valgum. **A** and **B**, Preoperative clinical and radiologic findings. Note the mechanical axis is traversing zone 2 bilaterally. Because the deformity was predominantly femoral, the femoral physis only was attacked. **C** and **D**, Radioclinical outcome at 1.9-year postimplant insertion. Note the mechanical axis of the left limb traverses the lateral half of the central one-third of tibia, i.e., optimal correction. Yet, the mechanical axis of the right limb traverses outside the central one-third (zone 1). Note the loosening backing up of the epiphyseal screws bilaterally. Both implants were removed, and patient was scheduled for implantation of the right medial tibial physis to correct residual valgus.

**Figure 3:**
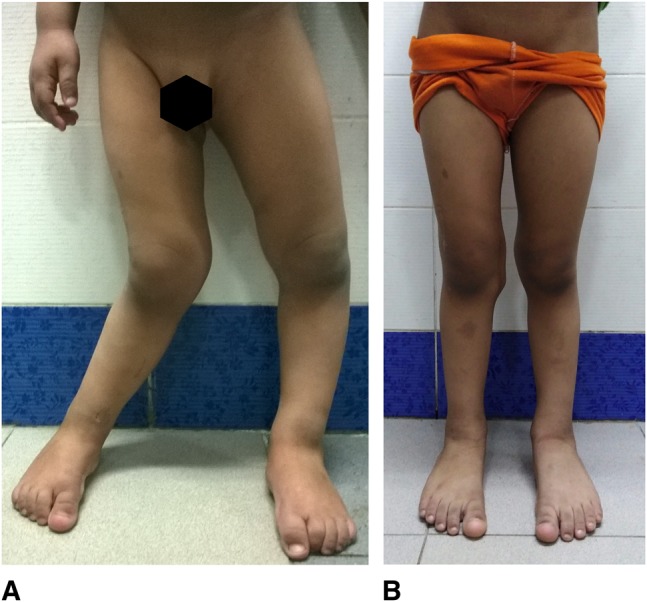
**A** and **B**, Guided growth for a 4-year-old boy with severe wind-swept deformity. **A**, Preoperative clinical appearance and (**B**) 1.7-year postoperative appearance immediately before implant removal shows optimal alignment.

**Figure 4:**
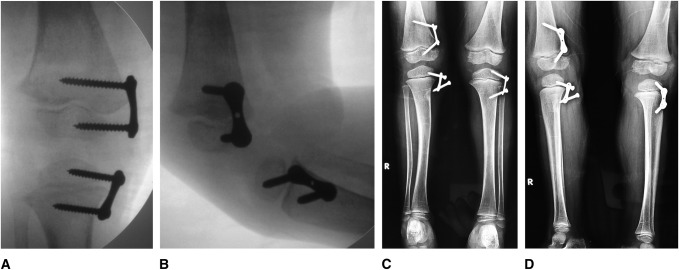
**A**–**D**, Radiographic appearance for the same patient as in Figure 3. **A** and **B**, Radiographs at time of implant insertion. **C** and **D**, Postoperative radiographic appearance (1.7 years). Despite massive implant disassembly especially of the tibial physis on the right limb with the severe genu valgum, full clinical correction was achieved.

The TFA, MAD, mLDFA, MPTA, and HEA demonstrated a highly statistically notable improvements at the final follow-up visit (Table 2). These radiographic improvements correlated positively with the clinical outcomes. Valgus knees demonstrated a significantly high speed of correction of TFA in contrast to varus knees (*P* = 0.034.) (Table 3). For both valgus and varus deformities, the speed of correction of TFA mounting to <30° and ≥30° was 1.1° per month (interquartile range 0.5 to 1.7) and 2.5°/month (interquartile range 2.1 to 3.0), respectively. This difference was highly significant (*P* < 0.001). MAD was expressed as percentage of half plateau width. Negative values were assigned for varus deformities, and positive values were assigned for valgus deformities. The preoperative MAD was −158.6 ± 75.1 and +146.9 ± 76.7 for varus and valgus knees, respectively. The values for the MAD were as follows: 51 knees (59%) acceptable alignment, 31 knees (36%) optimal alignment, and 4 knees (5%) overcorrected. The overall complication rate was small and tolerable and not necessarily disease or technique-related (Table 4 and Figures 2–4).

**Table 2:** Radiographic Outcomes at the Final Follow-up^a^

Factors	Preoperative		Postoperative		Paired *t*-test	
	Mean	SD	Mean	SD	*P* Value	Sig.
Varum						
Radiographic TFA	25.07	16.33	6.84	1.22	<0.001	S
mLDFA	103.50	11.11	88.75	1.24	<0.001	S
MPTA	79.50	7.86	87.95	0.96	<0.001	S
HEA	37.67	9.16	23.86	5.72	<0.001	S
Valgum						
Radiographic TFA	26.12	7.61	8.03	2.90	<0.001	S
mLDFA	80.76	7.71	88.56	2.58	<0.001	S
MPTA	94.35	6.61	87.41	2.89	<0.001	S
HEA	18.00	5.19	24.88	6.71	<0.001	S

HEA = Hilgenreiner-epiphyseal angle, MAD = mechanical axis deviation, it is expressed as percentage of half plateau width (negative values are assigned for varus deformities and positive values are assigned for valgus deformities), mLDFA = mechanical lateral distal femoral angle, MPTA = medial proximal tibial angle, NS = non-significant; TFA = tibiofemoral angle

a All measurements recorded in degrees.

**Table 3:** Duration of Plate Application and Speed of Correction as per Varus Versus Valgus Knees^a^

Factors	Deformity				*t*-test	
	Varum		Valgum			
	Mean	SD	Mean	SD	*P* Value	Sig.
Duration of correction (mo)	10.9	2.2	10.8	2.9	0.834^(T)^	NS
TFA correction	14.50	5–24	18.00	12–25	0.230^(M)^	NS
Rate of correction^b^	1.10	0.4–2.25	1.65	1.1–2.3	0.034^(M)^	S

TFA = tibiofemoral angle

a All measurements recorded in degrees; T = *t*-test; M = Mann-Whitney test.

b Rate of correction were measured by the amount of corrected TFA divided by the duration in months.

**Table 4:** Complications

Complication	Patients	Radioclinical Setting	Deformity (Knees)	Involved Physes/Hemiside	Management
Overcorrection	4 (5%)	Complication attributed to poor follow-up	2 valgus and 2 varus	2 medial femoral were overcorrected into varus, and 2 lateral tibial physes were overcorrected into valgus	Two femoral varus overcorrections were managed by reimplantation of another plate in the contralateral hemiside of the epiphysis until the desired correction was reached. The two tibial valgus overcorrections were simply put under observation as overcorrection was mild with no functional limitations.
Implant disassembly^a^	8 (16%)	All except one were epiphyseal screw loosening which were encountered in limbs that were corrected through one physis only and exhibited a TFA ≥ 30°^b^	3 valgus and 11 varus	3 medial femoral, 1 medial tibial, and 11 lateral tibial physes	Two of these patients (one physis each) achieved correction of the residual valgus and varus deformities through additional plating of the ipsilateral virgin medial tibial and lateral femoral physes, respectively, to speed up correction. The other 6 patients (13 physes) were followed up till full correction. Of the above 13 physes, 5 were bilateral genu varum, and one patient with a windswept deformity had 3 instrumented physes.
Screw breakage^a^	1 (2%)	Metaphyseal screw breakage. Consequently, absolutely no growth modulation was noticed around that knee.	1 varus	Occurred in right lateral tibial construct in a child with bilateral genu varum.	The left knee progressed to an acceptable correction of MAD. Both tibial implant constructs were removed, and the revision surgery was undertaken on the right knee only. The broken screw was exchanged with a new and longer one.
Additional surgery	4 (5%)	One tibia in a child with bilateral genu varum failed to derotate in conjugation with full correction of varus deformity.	3 varus and 1 valgus	3 lateral tibial and 1 medial femoral physis^c^	1 tibial derotation osteotomy. Tibial intoeing gait was markedly symptomatic to necessitate a derotation osteotomy. Also see 3 additional surgeries under implant disassembly (2) and screw breakage (1).
Persistent internal tibial torsion^d^	1 (2%)	One tibia in a child with bilateral genu varum failed to derotate in conjugation with full correction of varus deformity.	1 varus	1 lateral tibial	Tibial intoeing gait was completely asymptomatic. Patient was managed conservatively.
Rebound	1 (2%)	Rebound mounted to 50% of the clinical TFA recorded immediately after implant removal.	1 valgus	1 medial femoral	Rebound was associated with marked ligamentous collateral laxity and occurred shortly after implant removal.
Recurrence of rachitic activity	2 (4%)	The two patients of recurrence of rachitic activity occurred during the course of surgical treatment and before plate removal.	4 varus	4 lateral tibial	They were subjected to medical treatment until radiographic and biochemical healing. This did not affect the final outcome.
Superficial infections	2 (4%)	Infections were superficial and early onset.	1 varus and 1 valgus	1 medial femoral and 1 lateral tibial	Infections eventually resolved after implant removal. Infections had no effect on overall correction.

MAD = mechanical axis deviation, TFA = tibiofemoral angle

a Implant failure was subdivided into two categories namely implant disassembly or screw/plate breakage.

b The only exception was a complete extrusion of the epiphyseal and metaphyseal screw of the medial tibial physis in the genu valgum component of a windswept deformity. The ipsilateral femoral physis also suffered implant disassembly but to a lesser degree.

c These were the physes that were originally instrumented. Additional surgery to speed up correction was done on two lateral femoral and one medial tibial physes for varus and valgus knees, respectively, in addition to one derotation osteotomy.

d The 2 knees with persistent internal tibial torsion occurred in 51 knees with genu varum. It is noteworthy that knees with genu valgum did not originally exhibit internal tibial torsion.

## Discussion

### Surgical Indications and Outcomes

Predictors of spontaneous resolution of persistent severe lower-limb deformities despite successful medical therapy of nutritional rickets in regard to patient and disease characteristics are yet to be identified in the literature. In that regard, age and severity of deformity are crucial factors. Likewise, the exact indications of surgical interventions currently used to correct angular knee deformities in these children remain undetermined and mainly subject to surgeon's preference. Yet, a very recent prospective natural history study suggested that varum above 4 years and 18° of valgum above 9 years usually do not correct and may require surgical intervention.^24^ The indications for surgical intervention implemented in our study are fairly similar to those proposed by the above authors in regard to genu varum.^24^ In addition, we based our indications on clinical parameters such as symptomatic gait difficulties and deformity severity. It is noteworthy that this study^24^ was hospital-based which makes it specifically prone to bias. In addition, the study underestimated the role of environmental and genetic predisposition related to the pathoetiology of nutritional rickets.^23^ The fact that all but two tibial varus deformities with internal tibial torsion in our study have derotated in conjugation with varus correction indicates that tibial varus and internal torsion are strongly linked. The above natural history study^24^ failed to address the fate of internal tibial torsion known to be a close associate of varus. In our study, the markedly symptomatic tibial intoeing gait was used as one of the indicators of surgery in addition to its use as an outcome measure. Because of the dynamically developing TFA in childhood,^27^ we considered any mechanical axis traversing the central one-third of the widest diameter of the tibial epiphysis to be accepted. Yet, we opted for a more valgus orientation of the mechanical axis, ie, a mechanical axis traversing the lateral half of the central one-third. Long-term studies of guided growth around the knee suggested that varus alignment of the mechanical axis may be a precursor of rebound.^28^ The above argues for our stratification of radiographic outcomes. The rebound phenomena has also been attributed to children with a rapid rate of correction^29^ and to the underlying pathology.^30^ The radiographic findings of our study demonstrated that structural realignment of the MAD after guided growth surgery around the knee can have a notable positive effect on the hip posture in terms of measurement improvement of the HEA.

Most studies on guided growth surgery were retrospective and to some extent involved heterogeneous patient and disease cohorts in addition to diverse surgical methodologies and implants.^1,10,30,31^ In turn, this may undermine the capability of such studies to provide valid and generalizable conclusions. We presented the only homogeneous and prospective series on angular knee deformities in children with nutritional rickets. Various authors have reported satisfactory radioclinical outcomes in relatively small subsets of children with nutritional rickets.^1,31^ This goes in line with the overall outcome of our study.

### Complications and Evolved Surgical Strategy

**Figure 5:**
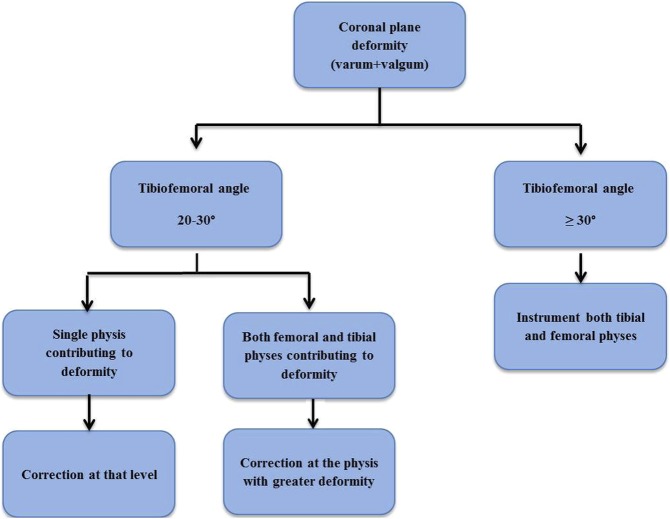
Final treatment algorithm. Surgery is indicated if the coronal tibiofemoral angle is ≥ 20° that is persistent or progressive over the past 6 months. If residual tibial torsion is deemed functionally symptomatic after reaching the desired correction in the coronal plane, a supramalleolar derotation osteotomy can be simultaneously pursued with implant removal.

Various studies reported screw loosening and have related it to a variety of factors namely, osteopenic bone quality such as resistant rickets, short screw length, and proximity of epiphyseal screw start point to physis.^2,9,32,33^ In consequence, some authors recommended longer and or larger screws to overcome such complications.^32,33^ We agree with the above authors as to the fundamental involvement of the epiphyseal screw in loosening. Yet, in our study, screw length was not an issue. The incidence of screw loosening or implant disassembly in our study was tolerable and mainly linked to degree of deformity and the overall duration of implant retainment from insertion to removal. Furthermore, this complication almost exclusively occurred in limbs where only one physis was implanted. This prompted us to upgrade our treatment protocol and attack both physes i.e., tibial and femoral—if the deformity mounted to TFA of ≥30° irrespective of the specific contribution of each physis to the overall MAD. This evolved strategy aimed at speeding the rate of correction thereby averting screw loosening. In the mild counterparts of limb deformity i.e., 20° to 30°, we achieved correction from the physis with greater contribution to deformity and neglected the other. We believe that minor distortions (10° to 15°) of the mLDFA or MPTA would be compensated as long as the mechanical axis is restored. The above authors managed this complication by a variety of methods namely screw removal, reinsertion of longer screws, conversion to an osteotomy, or simply observation.^2,32,33^ In our 14 knees (16%) which developed screw loosening, only 2 knees achieved correction of the residual valgus and varus deformities through additional plating of the ipsilateral virgin physis. In the remaining 12 knees, we simply chose to observe four of them until they achieved the end point for implant removal, and the remaining two were revised by guided growth. We acknowledge that screw loosening undermines the capability of the whole implant construct to provide growth modulation and cause undue delay in correction speed. Nevertheless, this observation strategy was successful in achieving an accepted correction at the expense of lengthy implant retainment. It is noteworthy that Masquijo et al^2^ in their series on failure of tension band plating recorded that three of nine physes had an overall time of implant retainment from insertion to removal of greater than 2 years with two implants retained for 4.5 years each. Yet, the authors failed to make any inferences from such data.^2^ This observation lends support to our explanation for screw loosening that incriminates a long implant retainment period as a likely causation. Our surgical strategy comprised drilling the near cortex for a few millimeters and then continuing screw insertion free handed to maximize screw purchase. In addition, we aimed at crossing the midpoint of the epiphysis and metaphysis at least. Longer screws were permissible as long as the far cortex was not violated. We estimated that this screw placement strategy would safeguard against implant disassembly. The upgraded and final treatment algorithm is depicted (Figure 5).

On the other hand, the incidence of screw breakage ranged from three physes (0.55%) in a series of 967 physes up to eight physes (26%) in a series of 31 physes.^1,11,34^ Funk et al^21^ reported an unacceptably high rate of implant failure (58%). Yet, they defined implant failure as implant migration or breakage.^21^ The studies that reported screw breakage strictly linked it with the metaphyseal screw in Blount disease with a severe deformity and high body mass index and to some extent linked with young age.^1,11,18,21,22,34^ In our study, we encountered one screw breakage of the right proximal medial tibial plate in a young child with bilateral genu varum. A large survey study^22^ reported that screw breakage occurred almost always in the metaphyseal screw-not where the head meets the shank but where the shank enters the lateral cortex. This was precisely the case in our only screw breakage case. The findings of the above-mentioned authors^1,11,18,21,22,34^ and our study outcomes point out that this screw breakage complication is an inherent characteristic of Blount disease and its underlying pathoetiology. We record one limb only with rebound phenomena.

### Study Limitations

We acknowledge limitations of this study. First, we relied on qualitative assessment (absent/present) of tibial torsion. We did not measure tibial torsion quantitatively per degrees of bimalleolar axis deviation with respect to the frontal plane nor did we resort to CT or MRI due to logistic challenges. It is noteworthy that the literature reports mixed results in regard to the accuracy of clinical bimalleolar axis measurement as an indicator for tibial torsion when measured against various standard reference tests.^35,36^ The satisfactory outcome of the parent-reported assessment regarding frequent falls and activity of daily life provides support for our qualitative measurement of the bimalleolar axis and corroborates our conclusions in regard to tibial torsion. Likewise, we assessed the rebound phenomenon clinically only due to suboptimal resource settings. Future studies should consider using more rigorous objective clinical and radiologic tools to assess tibial derotation. We did not correlate the age of the patient with the speed of correction as our patients belonged to a homogenous age group (young children < 5 years).

## Conclusion

The radioclinical outcome of this study demonstrates that guided growth surgery in young children with nutritional rickets is effective with a tolerable complication profile in the clinical setting described. Furthermore, these results expand with considerable certainty the range of indications in which guided growth can be practiced. Generally, tibial intoeing exhibited derotation with coronal plane correction irrespective of plate/screw positioning and derotation osteotomies are not deemed necessary unless there is a markedly troublesome gait and function. Our proposed algorithm for treatment of angular knee deformities in rachitic children may be used as a decision-taking guide for achieving the desired growth modulation in a more efficient manner. Generally, this algorithm may also have applicability to correction of coronal plane deformities caused by pathoetiologies other than rickets. The homogenous patient and disease characteristics allow for reasonable generalizability of the conclusions. Natural history studies are on great demand and may refine the indications of surgical intervention especially in regard to patient's age and deformity severity by providing prognostic insights.
